# Clearance of senescent cells enhances skin wound healing in type 2 diabetic mice

**DOI:** 10.7150/thno.100991

**Published:** 2024-08-26

**Authors:** Priyadarshani Nadeeshika Samarawickrama, Guiqin Zhang, Enfang Zhu, Xin Dong, Ayesha Nisar, Hong Zhu, Yuan Ma, Zheyan Zhou, Honglin Yang, Li Gui, Mei Cao, Wei Li, Yu Chang, Meiting Zi, Haoling Cui, Zhongping Duan, Xuan Zhang, Wen Li, Yonghan He

**Affiliations:** 1 Key Laboratory of Genetic Evolution & Animal Models, Kunming Institute of Zoology, Chinese Academy of Sciences, Kunming, Yunnan 650201, China.; 2 Key Laboratory of Healthy Aging Research of Yunnan Province, Kunming Institute of Zoology, Chinese Academy of Sciences, Kunming, Yunnan 650201, China.; 3 University of Chinese Academy of Sciences, Beijing 100049, China.; 4 Department of Endocrinology, the Second Affiliated Hospital of Dali University (the Third People's Hospital of Yunnan Province), Kunming, Yunnan 650011, China.; 5 Department of Orthopedics, the Third People's Hospital of Yunnan Province, Kunming, Yunnan 650011, China.; 6 Department of Pathology, the Third People's Hospital of Yunnan Province, Kunming, Yunnan 650011, China.; 7 Drug Discovery & Development Center, Shanghai Institute of Materia Medica, Chinese Academy of Sciences, Shanghai, China.

**Keywords:** cellular senescence, diabetic foot ulcer, imaging, senolytic, wound healing

## Abstract

**Background:** Diabetic foot ulcers (DFUs) pose a substantial healthcare challenge due to their high rates of morbidity, recurrence, disability, and mortality. Current DFU therapeutics continue to grapple with multiple limitations. Senescent cells (SnCs) have been found to have a beneficial effect on acute wound healing, however, their roles in chronic wounds, such as DFU, remain unclear.

**Methods and results:** We collected skin, fat, and muscle samples from clinical patients with DFU and lower limb fractures. RNA-sequencing combined with qPCR analyses on these samples demonstrate a significant accumulation of SnCs at DFU, as indicated by higher senescence markers (e.g., p16 and p21) and a senescence-associated secretory phenotype (SASP). We constructed a type 2 diabetic model of db/db mice, fed with a high-fat diet (Db-HFD), which were wounded using a 6 mm punch to the dorsal skin. HFD slightly affected wound healing in wild-type (WT) mice, but high glucose significantly delayed wound healing in the Db-HFD mice. We injected the mice with a previously developed fluorescent probe (XZ1208), which allows the detection of SnCs *in vivo*, and observed a strong senescence signal at the wound site of the Db-HFD mice. Contrary to the beneficial effects of SnCs in acute wound healing, our results demonstrated that clearance of SnCs using the senolytic compound ABT263 significantly accelerated wound healing in Db-HFD mice.

**Conclusion:** Collectively, these findings suggest that SnCs critically accumulate at wound sites, delaying the healing process in DFUs. Thus, targeting SnCs with senolytic therapy represents a promising approach for DFU treatment, potentially improving the quality of life for patients with DFUs.

## Introduction

Skin wound healing is a multifaceted process involving a delicate interplay between extracellular signals and cellular responses aimed at restoring tissue integrity and function 1. As the skin serves as a physical, chemical, and bacterial barrier, its capacity for wound healing is often regarded as a key indicator of both skin aging and overall health 2. Wounds are generally categorized into acute and chronic types based on various factors, such as duration and healing trajectory. Chronic wounds, particularly those associated with diabetes mellitus (DM), such as diabetic foot ulcers (DFUs), are marked by delayed healing and a propensity for infection, frequently leading to severe complications such as amputations, diminished quality of life, and increased mortality 3. The pathophysiology of impaired wound healing in diabetic patients is multifactorial, involving persistent inflammation, impaired angiogenesis, and dysfunctional cellular responses 3, 4. Existing therapeutic strategies for DFUs include glycemic control, enhancement of microcirculation, infection prevention, nutritional optimization, reduction of plantar pressure, wound debridement, *etc*. However, unfavorable outcomes, such as gangrene, lower-extremity amputation (LEA), and elevated mortality rates continue to pose a significant challenge 4. Consequently, a comprehensive understanding of the molecular and pathological mechanisms underlying DFUs is essential for the development of novel and effective therapeutic interventions.

An emerging focus in the study of DFUs is the role of cellular senescence 4. Cellular senescence is a state of irreversible cell-cycle arrest that results from extensive cellular replication 5, as well as from factors such as oxidative stress, genotoxic damage, oncogene activation and proteasome inhibition 6. Cellular senescence plays both physiological and pathological roles depending on the specific situation 7. For instance, cellular senescence acts as a mechanism for suppressing tumorigenesis 8. However, the excessive and aberrant accumulation of SnCs has been implicated in numerous age-related diseases, such as pulmonary fibrosis, liver damage, arteriosclerosis, retinopathy, osteoarthritis, dementia, and DM 6-11. Notably, the selective clearance of SnCs achieved through genetic strategy or pharmaceutical agents known as senolytics, has been shown to improve physical function, alleviate age-related pathologies and extend healthspan in aged mice 10, 12, 13. As of now, some senolytics have entered clinical trials and have shown promising effects 9. In the context of wound healing, SnCs can exert either beneficial or detrimental effects, depending on the specific physiological and pathological conditions 14, 15. Senescence has been shown beneficial effects in promoting tissue remodeling and regeneration, as evidenced by increased presence of SnCs during normal wound healing in mice 16. Additionally, cellular communication network factor 1 and 2 (CCN1 and CCN2) have been found to induce senescence in fibroblasts, thereby preventing excessive fibrosis 17, 18. However, in DFU patients, hyperglycemia may predispose cells to oxidative stress, mitochondrial dysfunction, and DNA damage, leading to the induction of cellular senescence at the wound site 4, 19. The presence of SnCs in this context may exacerbate inflammation and further disrupt the wound healing process 4. Despite these insights, the regulatory mechanisms governing cell senescence in DFUs are complex and the precise roles of SnCs in DFU healing remain unclear. We hypothesize that persistent accumulation of SnCs may contribute to delayed healing in the chronic wounds of diabetic patients, and that the clearance of SnCs with senolytics may facilitate wound healing by improving tissue function and promoting regeneration.

In this study, we revealed significant SnC accumulation across skin, fat and muscle tissues in DFU patients. Utilizing the near-infrared fluorescent probe XZ1208 20, we effectively traced the dynamic senescence during the process of wound healing in a diabetic mouse model. Notably, the clearance of SnCs using senolytic agent ABT263 resulted in accelerated wound healing in these diabetic mice. These findings indicate that more SnCs were induced at wound site and significantly affected wound healing in diabetic mice. Therefore, the targeted elimination of SnCs via senolytics may offer a promising therapeutic way for DFU wound healing in diabetic patients.

## Materials and methods

### Human wound sample collection

Seventeen non-DFU (nDFU) patients (age: 56.88±13.55 years) with lower limb fractures and fifteen type 2 DFU patients (age: 61.4±10.89 years) were recruited in this study. We collected samples below knee joints and active skin, fat, and muscle tissues around the wound after disinfection, deiodination, and debridement, ensuring the wound retained its complete structure. Each tissue area was 0.5 cm^2^ in size. We placed three different types of tissues into three transparent centrifuge tubes. The human samples were collected following protocols approved by the ethical review board at the Third People's Hospital of Yunnan Province (2023KY046). The study was conducted in accordance with the Declaration of Helsinki, and written informed consent was provided by all patients.

### RNA extraction, RNA-sequencing and data analysis

RNA extraction was carried out from all the nDFU and DFU patients, totaling 52 samples (n = 10 for skin, n = 8 for fat, and n = 8 for muscle), using TRIzol reagent (15596018CN, Thermo Fisher Scientific, MA, USA) in accordance with the manufacturer's protocol.

The polyA-enriched RNA-seq libraries were prepared for sequencing using the Illumina HiSeq 6000 platform by Biolinker Technology (Kunming) Co., Ltd. The human genomic data human hg38 build and gene annotation information were downloaded from the genecode database (gencodegenes.org). We mapped the clean reads onto human genome GRCh38 using the alignment software STAR V2.7.11b (https://github.com/alexdobin/STAR). Then we accurated transcript quantification from mapped data and calculated gene expression level using featureCounts V2.0.6 software (https://subread.sourceforge.net/featureCounts.html). We identified differentially expressed genes using the DESeq2 package (https://bioconductor.org/packages/release/bioc/html/DESeq2.html). Gene ontology biological process and pathway were analyzed by R package clusterProfiler and GSEA 21, 22. ggplot2 package was used for visualization of data.

### Animal experiment

#### Animals

The Institutional Animal Care and Committee of Kunming Institute of Zoology, Chinese Academy of Sciences approved the animal experimental protocols, and the animals were maintained according to the guidelines of the Animal Experimental Center of Kunming Institute of Zoology (approval number IACUC-RE-2024-01-002). We obtained male and female wild-type (WT) (C57BL/6) and Db (Lepr^-/-^) mice from Guangdong Biotechnology Co., Ltd. and housed them in the Animal Center of the Kunming Institute of Zoology, Chinese Academy of Sciences. We fed the mice a standard chow diet and a 6% high-fat diet (HFD) (Jiangsu Xietong Pharmaceutical Bio-engineering Co., Ltd., Nanjing, China), maintaining a 12 h dark cycle with unrestricted access to food and water for two months. After 12 h of fasting, blood samples were taken from the tail to measure blood glucose, which was confirmed to be above 13 mmol/L in the diabetic mice and below 10 mmol/L in the WT mice. Sinocare blood glucose test strips (GA-3, Kunming Diangong Technology Co. Ltd.) were used for blood glucose level measurements.

#### Wound model

At 16 weeks old, we anesthetized young Db and WT mice and wounded them. Mice were wounded using a 6 mm punch on the dorsal skin of Db and WT mice. The wounds were measured using a digital caliper and photographed. The wound areas were standardized by measuring the captured image. The wound area was determined by measuring its length (*L*) and width (W). Then the area was calculated using formula π × L/2 × W/2. The percentage of wound closure was calculated as follows: (day 0 area-day n area) / day 0 area × 100 (%).

#### Imaging

Mice were injected with 5 μM or indicated concentrations of senescence probe XZ1208 intravenously via the tail vein. XZ1208 is a near-infrared (NIR) fluorescent probe that specifically targets β-galactosidase (β-Gal), a well-established biomarker of cellular senescence. Upon injection into the tail vein, XZ1208 circulates in the bloodstream and subsequently penetrates cells, where it releases the fluorescent dye, enabling specific labeling of SnCs at the wound site while remaining inactive in non-SnCs 20. Compounds were dissolved in DMSO to obtain a 10 mM stock solution. Each mouse was injected with 5 μM compound formulated in 100 μL of solution containing 1% DMSO (1 μL of 10 mM stock solution), 2% TWEEN-80 (2 μL) (HY-Y1891, MedChemExpress, Shanghai, China) and 97% saline (97 μL). Mice were anesthetized using Zoletil 50 (5 mg/kg, Virbac, Carros, France) and then optical images were taken after 24 h at indicated time points using the IVIS Lumina XR (Caliper Life Sciences, Waltham, MA, USA). Quantification of images obtained from animals was performed using Living Image software 4.2 (Caliper Life Sciences, USA). Regions of interests (ROIs) were determined by choosing a square shape, and positive signals were measured and presented as signal intensities. All parameters were kept consistent for each experiment during data analyses. All experimental protocols were approved by the Animal Ethics Committee of the Kunming Institute of Zoology, Chinese Academy of Sciences. All procedures conformed to the principles of animal protection, welfare, and ethics and relevant national guidelines.

#### Mice with ABT263 treatment

Young WT mice were fed a normal diet (WT-ND) or a high-fat diet (WT-HFD), and Db mice were fed a high-fat diet (Db-HFD) (n = 8 each group). For senolytic treatment, mice were randomly assigned to one group of the Db-HFD group and intraperitoneally injected (IP) with ABT263 (0.1 mL/mouse, 40 mg/kg, q2d, five injections). ABT263 (GC12405, GlpBio Technology, CA, USA) was formulated in 10% DMSO, 40% polyethylene glycol 300 (HY-Y0873, MedChemExpress, Shanghai, China), 5% polysorbate (HY-Y1891, MedChemExpress), and 45% saline. The other three groups (WT-ND, WT-HFD, and Db-HFD) were intraperitoneally injected with vehicle (VEH) (0.1mL/mouse, q2d, five injections). Wound tissues were harvested for analysis on day 10 after receiving the last injection.

#### qPCR

Quantitative reverse transcription polymerase chain reaction (RT-qPCR) was employed to evaluate the mRNA expression levels of *p16, p21* and senescence-associated secretory phenotype (SASP) factors in human (n = 8 for nDFU and DFU) and mouse wound skin, fat and muscle isolated from WT-ND, WT-HFD, Db-HFD, and Db-HFD+ABT263 mice (randomly selected 6 samples per group). Total RNA was extracted from all samples using the TRIzol reagent (15596018CN, Thermo Fisher Scientific, MA, USA) according to the manufacturer's protocol. RNA purity and concentration were assessed spectrophotometrically. cDNA synthesis was performed using the reverse transcription kit (K1622, Thermo Fisher Scientific, MA, USA). Gene expression levels were quantified by qPCR using specific primers for the target genes and the SYBR Green master mix (TSE201, Tsingke, Beijing, China). The 2^-ΔΔCt^ method was employed to determine the relative mRNA expression levels, with GAPDH serving as the internal control.

#### Western blotting

Western blotting analysis was performed on total protein extracts isolated from human (n = 4 for nDFU and DFU, respectively), and mouse wound skin, fat and muscle isolated from WT-ND, WT-HFD, Db-HFD and Db-HFD+ABT263 (n = 3 randomly selected tissue samples per group) using RIPA buffer (BP-115DG, Boston BioProducts, Inc., USA) supplemented with phenylmethanesulfonyl fluoride (PMSF) solution (ST507, Beyotime Biotech Inc., Beijing, China). Protein concentration was determined using a bicinchoninic acid (BCA) protein assay kit (P0010, Beyotime Biotech Inc., Beijing, China). Equal amounts of protein lysates were resolved by sodium dodecyl sulfate-polyacrylamide gel electrophoresis (SDS-PAGE) (A1010, Solarbio Life Science, Beijing, China) and transferred onto PVDF membranes. The membranes were subsequently blocked with 5% non-fat dry milk and incubated overnight at 4 °C with primary antibodies p16 (ab51243, Abcam, Cambridge, UK), p21 (2947, Cell Signaling Technology, Danvers, MA, USA). Following incubation with appropriate secondary antibodies, protein bands were visualized using an enhanced chemiluminescence (ECL) detection system. ImageJ software was used to quantify protein expression levels, normalized to β-actin as a loading control.

#### H&E staining

Histological analysis of human and mouse skin wound tissue sections was performed using hematoxylin and eosin (H&E) staining. Skin tissue samples were first fixed in 10% paraformaldehyde to preserve tissue architecture. Subsequently, the fixed tissues underwent deparaffinization and rehydration steps to remove paraffin embedding medium and restore tissue hydration, facilitating optimal staining solution (Harris) (BA4025, Baso Diagnostics, Zhuhai, China). Following these preparatory steps, the tissues were stained with H&E to visualize nuclei and cytoplasmic components, respectively. Stained tissue sections were then examined using an Axio Observer microscope equipped with the Airyscan platform (Carl Zeiss Meditec AG, Jena, Germany) for high-resolution imaging.

### Statistical analysis

All statistical analyses were performed and figures were drawn using GraphPad Prism v8. All data are presented as means ± standard error of the mean (SEM). Comparisons were made using two-tailed unpaired t-test when comparing two experimental groups. For Student's t-tests that failed the normality test, the Mann Whitney test was used. For comparisons between more than two groups, one-way analysis of variance (ANOVA) with Tukey's or Dunnett's post hoc test was used. Age distribution was analyzed by Chi-square test. *p* < 0.05 was considered significant.

## Results

### RNA-sequencing data suggest accumulation of senescent cells in the skin, fat and muscles of DFU patients

**Figure 1:**
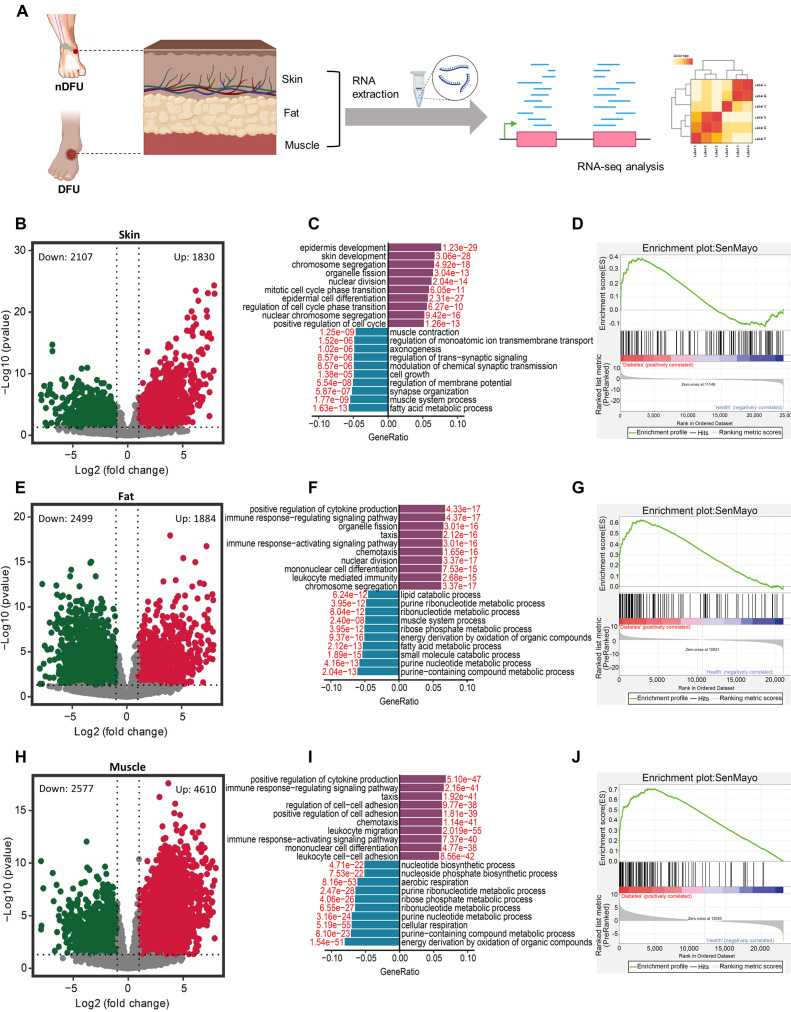
** Differential gene expression and pathway analysis in the skin, fat, and muscle tissues between nDFU and DFU patients. A**, Schematic diagram illustrating human wound sample collection and data analyses. **B**, Volcano plot showing DEGs in the skin between the nDFU and DFU patients. **C**, Bar plot shows enrichment of biological processes for DEGs in the skin between nDFU and DFU patients. **D**, Gene set enrichment analysis of SenMayo pathways in nDFU and DFU skin tissues (n = 10) respectively. **E**, Volcano plot showing DEGs in the fat between the nDFU and DFU patients. **F**, Bar plot shows enrichment of biological processes for DEGs in the fat between nDFU and DFU patients. **G**, Gene set enrichment analysis of SenMayo pathways in nDFU and DFU fat tissues (n = 8) respectively. **H,** Volcano plot shows DEGs in the muscle between the nDFU and DFU patients. **I,** Bar plot shows enrichment of biological processes for DEGs in the muscle between nDFU and DFU patients. **J,** Gene set enrichment analysis of SenMayo pathways in nDFU and DFU muscle tissues (n = 8) respectively. In **B**, **E**, **H**, red points represent upregulated DEGs; blue points represent downregulated DEGs; gray points represent unchanged genes. In **C**, **F** and **I**, purple bars represent upregulated GO terms, blue bars represent downregulated GO terms, and the red numbers indicate the adjusted *p* values. nDFU, non-diabetic foot ulcer; DFU, diabetic foot ulcer; DEGs, differentially expressed genes; GO, gene ontology.

DFUs infiltrate the skin, subcutaneous fat, and muscle compared to the normal skin in nDFU patients (Figure S1A-D). It has been reported that SnCs accumulate in the skin of DFU patients 23. To determine the senescence status across tissues, we collected skin, fat, and muscle samples from clinical patients with DFU. Meanwhile, we collected same tissues from the wound site of age-matched patients with lower limb fractures as the nDFU (Figure 1A). The information of the nDFU and DFU patients was shown in Table 1. There was no difference in age, sex, white blood cells (WBC), neutrophil, lymphocyte, aspartate aminotransferase (AST), alanine aminotransferase (ALT), urea nitrogen, serum creatinine, uric acid, low-density lipoprotein cholesterol (LDL-C) and total cholesterol (TC) between the nDFU and DFU groups. Compared to the nDFU group, DFU patients had higher levels of blood glucose, ultrasensitive C-reactive protein, and platelet count, but lower levels of red blood cells (RBC), hemoglobin, albumin and high-density lipoprotein cholesterol (HDL-C).

Performing RNA-sequencing on the samples, we identified 3937 differentially-expressed genes (DEGs), with 1830 upregulated and 2107 downregulated, in skin tissues between the nDFU and DFU groups (Figure 1B). The 1830 upregulated DEGs in the DFU were enriched in skin development and proliferation-related terms, such as chromosome segregation, organelle fission, nuclear division, and mitotic cell cycle phase transition, suggesting that cell renewal was very active at the wound site. In contrast, the 2107 downregulated DEGs were mainly enriched in metabolism (e.g., fatty acid metabolic process, regulation of member potential, *etc*.) and cell growth (Figure 1C). To test the senescence of skin, we extracted the SenMayo genes 24, and compared their expression between the nDFU and DFU groups. As shown in Figure 1D, many SenMayo genes were found in the DEGs of DFU skin, with a high enrichment score, suggesting the accumulation of SnCs in the skin of DFU patients.

In fat tissues, there were 4383 DEGs in the DFU group compared to the nDFU group, among which 1884 were upregulated and 2499 were downregulated (Figure 1E). The upregulated DEGs were enriched in terms such as positive regulation of cytokine production, immune response-regulating signaling pathway, and chemotaxis, *etc*., while the downregulated were enriched in metabolism, such as purine nucleotide, fatty acid metabolism, ribonucleotide and lipid catabolic metabolism (Figure 1F). Similarly, the SenMayo genes were enriched in DFU fat tissues (Figure 1G).

In muscle tissues, we identified 7187 DEGs with 4610 upregulated and 2577 downregulated (Figure 1H). Very similar to the enrichments in fat tissues, the upregulated DEGs in the muscle were enriched in cytokine production, immune response, chemotaxis (Figure 1I). Other enrichments included cell adhesion, immune cell differentiation and migration. The downregulated DEGs were mainly enriched in metabolism, such as purine nucleotide, ribonucleotide, ribose phosphate, aerobic, *etc*. (Figure 1I). As shown in Figure 1J, most of the SenMayo genes were also enriched in DFU skin muscle tissues.

Above all, RNA-sequencing analysis suggest that SnCs accumulate across skin, fat and muscle tissues in the DFU patients. The downregulated DEGs in the 3 tissues share similarities in enrichment, that is, energy metabolism. Skin displayed a different expression pattern for the upregulated DEGs compared to fat and muscle tissues which showed higher levels of cytokines and chemokines, representing the major component of SASP.

### Validation of senesce markers in skin, fat and muscles of DFU patients

**Figure 2:**
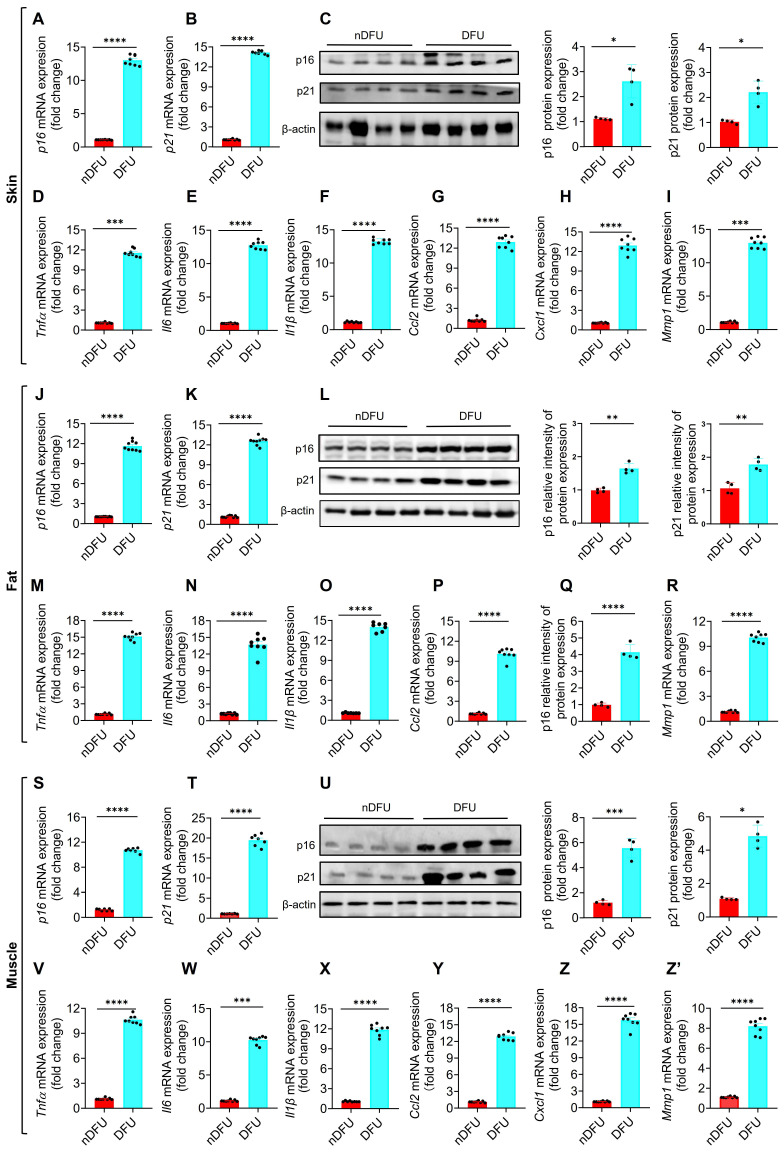
**Senescence markers in the skin, fat, and muscle of nDFU and DFU patients. A-C,** mRNA and protein levels of *p16* and *p21* in the wound skin were detected by qPCR (n = 6) and Western blotting analysis (n = 4), respectively. **D-I,** SASP factors (*Tnfα, Il6, Il1β, Ccl2, Cxcl1, and Mmp1*) in the wound skin were detected by qPCR (n = 6). **J-L,** mRNA and protein levels of p16 and p21 in the wound fat were detected by qPCR (n = 6) and Western blotting analysis (n = 4 for each group), respectively. **M-R,** SASP factors in the wound fat were detected by qPCR (n = 6). **S-U,** mRNA and protein levels of p16 and p21 in the wound muscle were detected by qPCR (n = 6) and Western blotting analysis (n = 4), respectively. **V-Z,** SASP factors in the wound muscle were detected by qPCR (n = 6). Data were analyzed by two-sided Student's t-test and presented as Mean ± SEM. nDFU, non-diabetic foot ulcer; DFU, diabetic foot ulcer; DEGs. *p < 0.05, **p < 0.01, ***p < 0.001, and ****p < 0.0001 compared to nDFU group.

To validate the accumulation of SnCs in DFU samples (>55 years), we first tested* p16* and *p21*, two major senescence markers of cellular senescence 6. The mRNA and protein levels of p16 and p21 were significantly higher in the skin of DFU group compared to the nDFU group (Figure 2A-C). The major SASP factors, including *Tnfα*, *Il6*, *Il1β*, *Ccl2*, *Ccl8*, *Cxcl1, Cxcl2, Cxcl3* and *Mmp1*, were also increased in the DFU skin (Figure 2D-I; Figure S1E-G). Among them, *Tnfα*, *Il6*, and *Il1β* are major proinflammatory factors, *Ccl2, Ccl8*, *Cxcl1, Cxcl2, and Cxcl3* are chemokines, and *Mmp1* is a matrix metallopeptidase. Same results were got in fat (Figure 2J-R; Figure S1H-J) and muscle tissues (Figure 2S-Z'; Figure S1K-M). Compared to the younger nDFU patients (35-50 years), there were no differences or just a slight increase in senescence markers in the older nDFU patients (>55 years) (Figure S2). Additionally, we assessed senescence markers in younger nDFU and DFU patients (35-50 years) and observed elevated expression levels of these markers in the skin, fat, and muscles of DFU patients compared to nDFU patients (Figure. S3). These findings validated that SnCs accumulate significantly across tissues at the wound site of DFU patients, independent of age, and hyperglycemia is likely the major trigger of senescence.

### Delayed wound healing was associated with SnCs in the skin of diabetic mice

**Figure 3:**
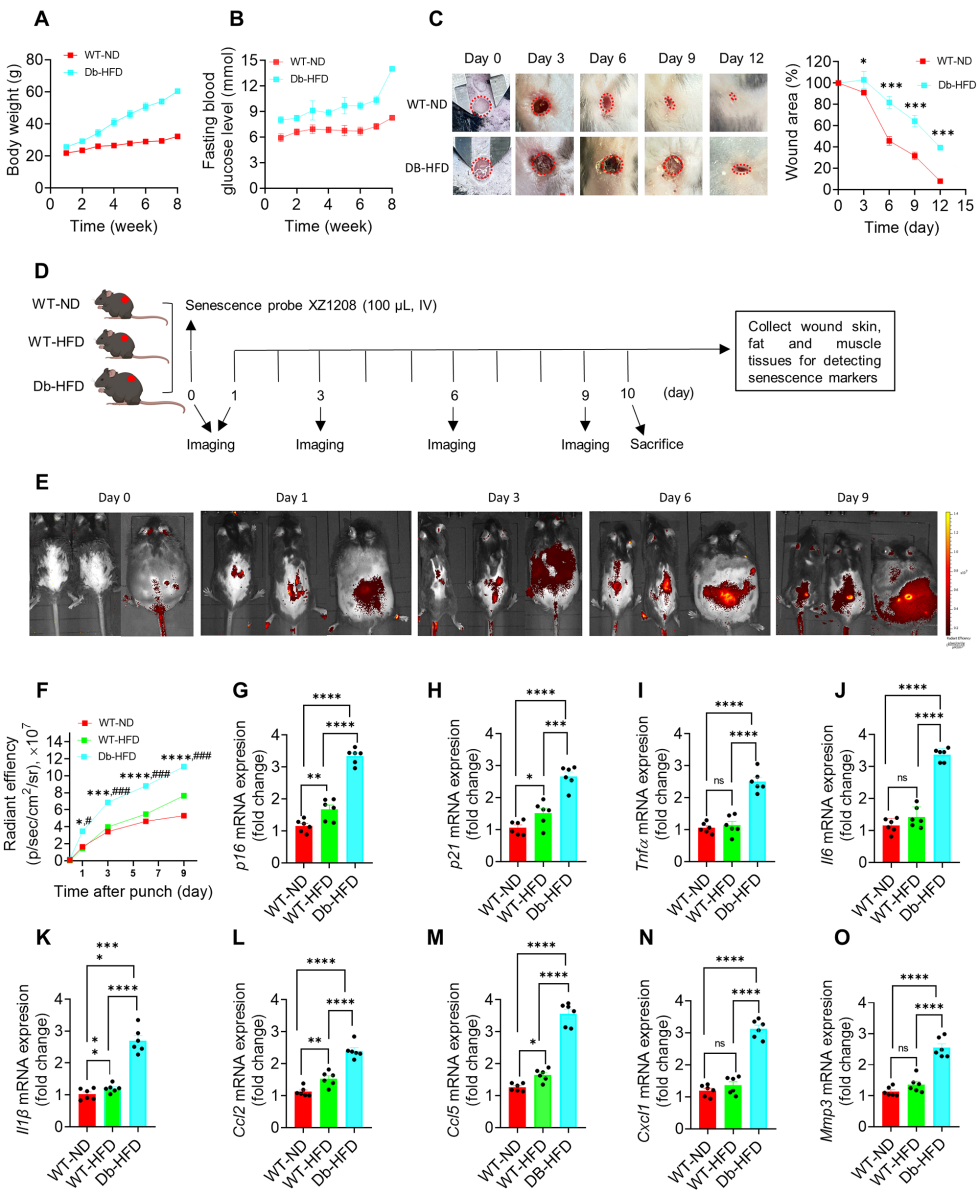
**Delayed healing in diabetic wound was associated with cellular senescence. A,** Body weight and **B,** fasting blood glucose levels in WT-ND and Db-HFD mice. **C,** The dorsal skin of mice was wounded using 6 mm punches, followed by a scale of the wound area in WT-ND and Db-HFD mice at the indicated time points (n = 7). The skin wound area was measured and expressed as a percentage of the initial wound area. **D,** Illustration of experimental design. The dorsal skin of mice was wounded using 6 mm punches, and were injected 100 μL XZ1208 intravenously via the tail vein (day 0) and imaged at indicated time points. Mice were euthanized to harvest wound tissues for analysis. **E,** Mice were imaged using the IVIS imaging system at indicated time points. **F,** Quantification of fluorescence signals in **E**. **G-O,** mRNA levels of *p16*, *p21*, and SASP factors (*Tnfα, Il6, Il1β, Ccl2, Ccl5, Cxcl1, Mmp3*) in wound skin on day 9 after the punch (n = 7). Data were analyzed by one-way ANOVA and presented as Mean ± SEM. WT-ND, wild type mice fed a normal diet; Db-HFD, Db mice fed a high-fat diet. *p < 0.05, **p < 0.01, ***p < 0.001, and ****p < 0.0001 in the indicated comparisons.

To test wound healing rate in diabetes and non-diabetes, we constructed type 2 diabetic mice by Db-HFD. After 2-month feeding, the Db-HFD mice gained more body weight than the WT-ND mice (Figure 3A). Fasting blood glucose levels were more than 13 mmol/L in the Db-HFD mice, but less than 10 mmol/L in the WT-ND group (Figure 3B), suggesting that the diabetic mouse model was successfully constructed. We gave the mice a 6 mm punch to the dorsal skin, and monitored the wound healing on day 0, 3, 6, 9 and 12. We observed a significant difference in the wound area between the WT-ND and Db-HFD mice from day 3 after punch, and more obvious on day 6, 9 and 12 (Figure 3C; Figure S4). On day 12, the wound was almost healed in the WT-ND mice, but still unhealed in the Db-HFD mice (Figure 3C; Figure S4). This result suggests that the diabetic mice had slower wound healing rate than the normal mice, which may mimic the situation in nDFU and DFU patients in clinic.

To check the presence of SnCs in the mouse model, we created identical punch wounds in another batch of mice and measured a series of senescence markers (Figure 3D). As the diabetic mouse model was induced by HFD, the effect of HFD on senescence induction in the WT mice was also tested. Here we used our previously developed senescence-detecting probe XZ1208 to test senescence-associated beta-galactosidase (SA-β-gal), one of the most widely used markers for SnC detection 25. XZ1208 can be cleaved rapidly by SA-β-gal and produces a strong fluorescent signal in SnCs, allowing the detection of SnCs *in vitro* and *in vivo* 20. As shown in Figure 3E-F, HFD feeding did not increase the fluorescent signal on day 1, 3, and 6, but slightly increased on day 9. In contrast, Db-HFD mice displayed a significant increase in the fluorescent signal from day 1 to day 9 (Figure 3E-F), suggesting that a single HFD slightly induced senescence in WT mice, but substantially induced senescence in the Db mice. Consistently, we tested other senescence markers including *p16*, *p21*, and SASP factors, and found their gene expression levels were slightly increased in the WT-HFD mice, but strikingly increased in the skin, fat and muscle of Db-HFD mice (Figure 3G-O; Figure S5A-R), suggesting diabetic mice were easier to accumulate SnCs after receiving skin punch. These findings indicate that delayed wound healing was associated with SnCs accumulation in the skin of diabetic mice.

### Clearance of SnCs accelerates skin wound healing in type 2 diabetic mice

**Figure 4:**
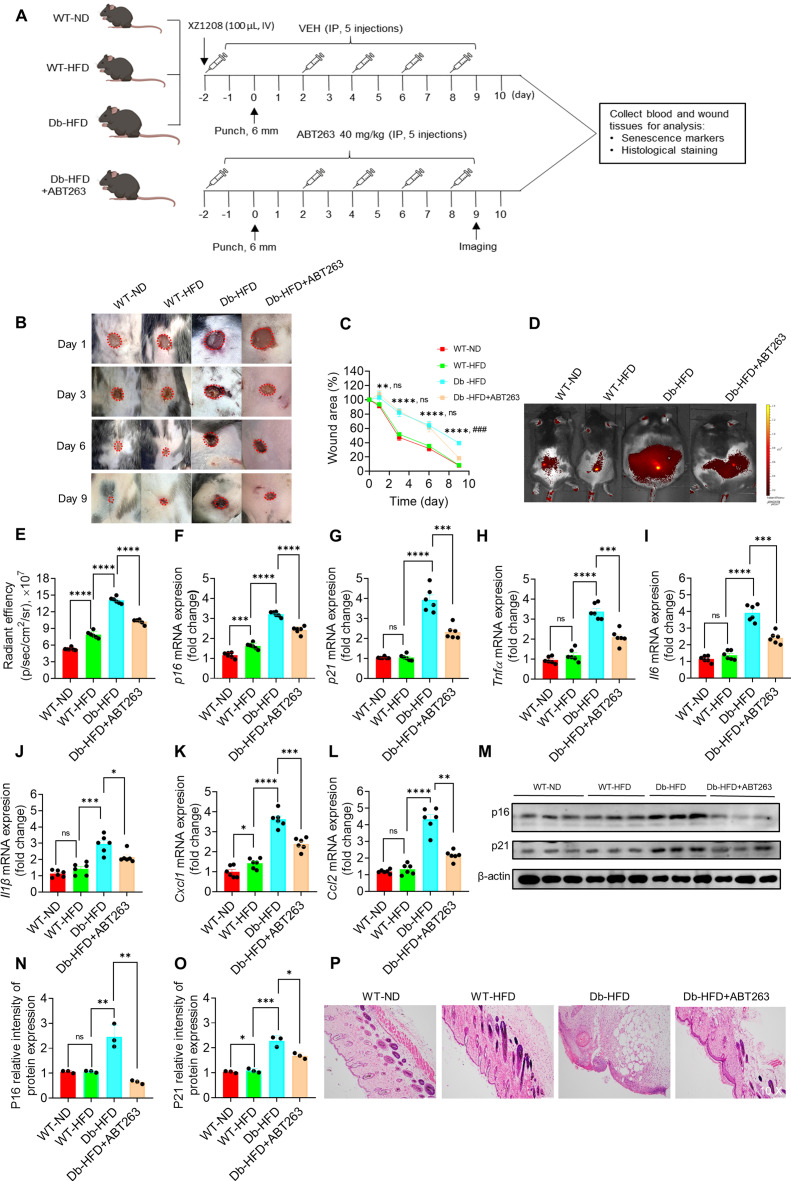
**Clearance of SnCs with ABT263 accelerated wound healing in diabetic mice**. **A,** Illustration of experimental design. The dorsal skin of mice was wounded using 6 mm punches. Mice in one of the Db-HFD groups received 40 mg/kg ABT263 via IP two days before punch, and were treated every 2 days (q2d) for a total of five injections. Mice in the other groups were treated with VEH. Mice were injected with 100 μL XZ1208 intravenously via the tail vein two days prior to the punch and scale of the wound area (day 1, 3, 6, 9). Mice were imaged on day 9. Mice were euthanized to harvest wound tissues for analysis 24 h after imaging. **B-C,** Representative skin wound area in the mice of each group at the indicated time points. The wound area was expressed as a percentage of the initial wound area (n = 8, 8, 7, 6 for WT-ND, WT-HFD, Db-HFD and Db-HFD+ABT263 groups, respectively). *Db-HFD verus WT-ND; ^ns^Db-HFD verus Db-HFD+ABT263 on day 1, 3, and 6; ^#^Db-HFD verus Db-HFD+ABT263 on day 9. **D,** Six mice were randomly selected for imaging using the IVIS imaging system on day 9. **E,** Quantification of fluorescence signals in D. **F-L,** *p16, p21* and SASP factors (*Tnfα, Il6, Il1β, Cxcl1, Ccl2*) were detected by qPCR (n=6 for each group). **M-O,** p16 and p21 protein levels on skin wound tissues were detected by Western blotting (n = 3). **P,** HE staining of skin wounds on day 10. The data are analyzed by one-way ANOVA and presented as Mean ± SEM. *p < 0.05, **p < 0.01, ***p < 0.001 and ****p < 0.0001 in the indicated comparisons.

Cellular senescence is essential for wound healing in acute wound 16, but its role remains unclear in wounds of chronic diseases, such as DFU. We previously proposed that clearance of SnCs in DFU may help wound healing 4. Here we administrated the mice with ABT263, a compound that selectively clears SnCs 20, 26, 27, to test the role of SnCs in wound healing of Db-HDF mice (Figure 4A). HDF feeding slightly increased the body weight and fasting glucose in WT-HFD mice, but strikingly increased their levels in the Db-HFD mice (Figure S6A-B). Two days before the punch, we started to administrated the mice with ABT263 and senescence probe XZ1208, and continued ABT263 dosing on day 2, 4, 6 and 8 (Figure 4A). The long-term labeling effect of XZ1208 20 allows us to detect the clearance effect on SnCs on day 9. As shown in Figure 4B-C, wound area in the Db-HFD groups was greater than the WT-ND or WT-HFD groups on day 1, 3, and 6. There were not any differences between the WT-ND and WT-HFD groups, suggesting that HFD did not significantly affect wound healing in the WT mice (Figure 4B-C; Figure S6C). Notably, we observed that ABT263 treatment accelerated skin wound healing in the Db-HFD mice compared to the Db-HDF mice (Figure 4B-C; Figure S6C). These observations suggest that ABT263 treatment helps wound healing the diabetic mice.

To verify whether ABT263 can clear SnCs in the mouse skin after punch, we tested senescence markers with senescence probe XZ1208, qPCR and Western blotting. Consistent with above results (Figure 3E-O; Figure S5A-R), we found that HFD slightly increased some senescence markers in the WT mice, but significantly increased the senescence markers in Db mice (Figure 4D-O). Notably, ABT263 treatment can reduce the senescence markers in the Db-HFD mice, including fluorescent signals (Figure 4D-E), mRNA levels of *p16*, *p21* and SASP factors (Figure 4F-L). We further validated the effect of ABT263 on protein levels of p16 and p21 (Figure 4M-O). HE staining also supported the role of ABT263 on skin wound healing, showing decreased inflammation and well-remodeling of intact skin structure (Figure 4P). All in sum, these findings clearly show that clearance of SnCs in the skin is beneficial for wound healing in diabetic mice.

## Discussion

This study elucidates the pivotal role of SnCs in the impaired wound healing observed in DFUs and demonstrates that the targeted clearance of these cells using senolytic compounds can significantly accelerate healing in a diabetic mouse model. These findings contribute to a growing body of evidence indicating that cellular senescence is a double-edged sword in wound healing, beneficial in acute wounds but detrimental in chronic conditions such as DFUs 15. Recent research supports this duality, showing that *p21-*positive fibroblasts delay wound healing in aged mice but improve when *p21* expression is inhibited 28.

Similarly, findings from another study indicate that while *p21/p53* expression aids wound healing in young skin, its suppression in the elderly leads to delays 29. Wound healing typically proceeds through dynamic and interactive stages: hemostasis, inflammation, proliferation, and remodeling, which partially overlap 30, 31. SnCs play crucial role in these processes, contributing favorably by promoting extracellular matrix (ECM) deposition and epithelialization, as well as regulating tissue remodeling, fibrosis, and inflammation 17, 32, 33. However, in DFUs, the persistent accumulation of SnCs leads to chronic inflammation and impaired wound healing.

The RNA-sequencing and qPCR analyses in our study reveal a marked accumulation of SnCs in the skin, fat, and muscle tissues of DFU patients. The elevated expression of senescence markers *p16* and *p21*, alongside the increased presence of SASP factors such as *Tnfα*, *Il6*, and *Il1β* underscores the chronic inflammatory state induced by senescence. This state contributes to the persistent wound-healing impairment in DFUs, aligning with previous studies linking cellular senescence to various chronic diseases. For instance, it has been noted that SnCs in diabetic ulcers exacerbate inflammation and inhibit cell proliferation 34, 35, mirroring our findings in DFU patients. Using the Db-HFD mice, we confirmed that hyperglycemia and a high-fat diet synergistically delay wound healing. The increased SnC accumulation, evidenced by higher senescence signals and elevated levels of *p16, p21*, and SASP factors in Db-HFD mice, mirrors the clinical scenario in DFU patients. This delayed wound healing in diabetic mice substantiates the hypothesis that SnCs play a critical role in the chronicity of diabetic wounds, as reported in recent studies 36, 37.

The most compelling finding of our study is the accelerated wound healing observed in Db-HFD mice following treatment with the senolytic compound ABT263. This result underscores the therapeutic potential of senolytics in managing chronic wounds. ABT263 effectively reduced the senescence markers and SASP factors, leading to improved tissue remodeling and decreased inflammation. These findings suggest that senolytic therapy not only targets SnCs but also ameliorates the adverse microenvironment they create, thus promoting more effective wound healing. Previous studies have shown that the clearance of SnCs via ferroptosis faciliated diabetic wound healing, further supporting this therapeutic approach 35, 38, 39. Clearance of SnCs has also been demonstrated to delay aging and extend healthspan 10, 12, 40, 41. Given the significant morbidity and mortality associated with DFUs, our results offer a promising avenue for developing new treatments. The ability of senolytics to clear SnCs and enhance wound healing could transform DFU management, shifting the focus towards addressing underlying cellular and molecular dysfunctions rather than solely treating symptoms. Beyond ABT263, several novel techniques are being explored for the senolytic treatment of diabetic wounds. For example, Fe_3_O_4_ nanospheres were encapsulated with galactose-modified poly (lactic-co-glycolic acid) (PLGA), which selectively eliminated SnCs by triggering ferroptosis of SnCs 39. Zhao *et al*. utilized poly-l-lysine/sodium alginate (PLS) modified with tarabostat (PT100) and encapsulated the PARP1 plasmid for delivery to target the dipeptidyl peptidase 4 (DPP4) receptor, eliminating senescent fibroblasts and reducing SASP, which in turn promoted wound healing in diabetic mice 42. Compared to DDP4, Bcl-xL is highly expressed in the skin, fat and muscle tissues (https://www.proteinatlas.org/), suggesting that targeting Bcl-xL with ABT263 may be more effective in clearing SnCs at the wound site of skin. Although these findings underscore the potential benefits of reducing SnCs in promoting diabetic wound healing, none of these therapeutic approaches have been tested in clinic patients. To date, only three senolytics have been investigated in clinical trials (https://clinicaltrials.gov/), the combination of dasatinib and quercetin (D+Q), fesetin, and UBX1325, which are conducted to test the therapeutic potential in idiopathic pulmonary fibrosis (IPF), cognitive impairment, osteoarthritis, or vascular function, however, none have been tested for DFUs in either preclinical or clinical studies. Additionally, other innovative approaches, including bioactive inorganic particles-based biomaterials 43, hydrogel biomaterials 44, MiR-17-5p-engineered small extracellular vesicles (sEVs) 45, small interfering RNA (siRNA) 36, and senomorphic compounds 4 have been reported to enhance apply diabetic wound healing. Future clinical trials are necessary to validate the efficacy and safety of these senolytics, techniques, and materials in human patients with DFUs, as emphasized by recent studies highlighting the potential translational impact of these approaches 2, 4, 46.

Moreover, the differential impact of SnCs in acute versus chronic wounds warrants further investigation to delineate the context-dependent roles of senescence. Understanding the regulatory mechanisms governing SnC accumulation and SASP factor release in diabetic wounds could lead to the development of more targeted and effective therapies. Recent research underscores the complexity of senescence regulation and its varied impact on different tissues, highlighting the need for tailored therapeutic approaches 47, 48. SnCs in wound healing are transient in fibroblasts 16, 37 and keratinocytes 49. Transient exposure to SASP factors increases the expression of stem cell-related genes in senescent keratinocytes, enhancing skin regenerative capacity. However, prolonged exposure leads to cell-intrinsic senescence arrest to counteract continuous regenerative stimuli 49. CCN1 and cytochrome P450 61 (CYP61) are dynamically expressed at wound repair sites and can induce fibroblast senescence through integrin α6β1 and heparan sulfate proteoglycans. CCN1-induced senescent fibroblasts accumulate in granulation tissues and express antifibrotic genes 17. Additionally, highly concentrated trehalose induces SA-β-gal activity in fibroblasts via the p21 pathway, promoting angiogenesis and keratinocyte proliferation, thus enhancing wound repair 50. DFUs are representative of chronic wounds, which also include pressure ulcers, vascular ulcers, radiation ulcers, *etc*. Like DFUs, these chronic wounds can persist for extended periods without healing and may lead to amputation if not adequately managed. Various therapies, such as wound debridement, anti-inflammation, local anesthesia, negative-pressure therapy, hyperbaric oxygen therapy, stem cell transplantation, tibial cortex transverse transport, and engineered skin, have been used to treat chronic wounds, but many of them continue to grapple with limitations or less effective 4, 51-53. Since cellular senescence plays an important role in developing DFUs, and clearance of SnCs promotes DFUs healing, we suggest that senolytics may be applicable for other chronic wounds.

While targeting cellular senescence presents a promising strategy for targeting chronic wounds, it is important to recognize that clearance of SnCs may raise safety concerns. SnCs can be beneficial under certain conditions 54, such as tissue regeneration, development, tumor suppression, insulin secretion, *etc*. Consequently, their clearance could potentially have detrimental effects on health. For example, the continuous or acute removal of senescent vascular endothelial cells in the liver disrupted blood-tissue barriers and resulted in perivascular fibrosis in various tissues; eliminating SnCs have been found to exacerbate pulmonary hypertension in mice 55; and the pharmacological or genetic ablation of senescent hepatic stellate cells impairs liver regeneration 56. SnCs may recruit immune cells through the SASP and promote tissue repair and remodeling. Over-clearance of SnCs could impair the recruitment of immune cells to clear SnCs. Furthermore, SnCs are heterogeneous and dynamic, shifting with time and with changes in the tissue microenvironment. Diabetic patients concurrently contend with various complications, *e.g.*, chronic kidney disease, chronic heart failure, diabetic nephropathy, diabetic peripheral vasculopathy, metabolic and nutritional disturbances, *etc*. 3, 4, 57-60. Therefore, it is important to monitor patient symptoms to reduce or avoid any unexpected outcomes when treating DFU patients with senolytics. Above all, effective therapies, including senolytics, will need precise dosage and administration to clear pathologic SnCs while sparing healthy SnCs to reduce potential side effects on health 54.

In conclusion, this study provides compelling evidence that SnCs contribute to the impaired wound healing seen in DFUs and that their targeted clearance via senolytic compounds represents a promising therapeutic strategy. Our findings not only advance the understanding of the pathophysiology of diabetic wounds but also open new avenues for therapeutic intervention, potentially improving the quality of life for patients suffering from this debilitating condition.

## Supplementary Material

Supplementary figures.

## Figures and Tables

**Table 1:** Clinical characteristics of diabetic foot ulcer (DFU) and non-diabetic (nDFU) patients.

Variables	DFU (n = 15)	nDFU (n =17)	*p* value
Gender (M/F)	9/6	9/8	0.6879
Age (years)	61.4±10.89	56.88±13.55	0.3114
Blood glucose (mmol/L)^*^	10.08±3.93	5.12±1.32	0.0002
WBC count (10^9^/L)	10.59±5.78	8.03±2.31	0.1253
Neutrophil count (10^9^/L)	7.97±5.7	5.73±2.41	0.1748
Lymphocyte count (10^9^/L)	1.81±0.74	1.66±0.67	0.5367
Neutrophil rate (%)	71.24±11.44	69.21±11.96	0.6278
Lymphocyte rate (%)	20.41±9.72	22.53±10.56	0.5603
Ultrasensitive C-reactive protein (mg/L)*	65.64±55.26	8.23±10.12	0.0013
Hemoglobin (g/L)*	106.87±22.27	135.29±17.09	0.0003
RBC count (10^12^/L)*	3.78±0.77	4.44±0.5	0.0063
Platelet count (10^9^/L)*	379.33±133.04	237.94±81.99	0.0009
Aspartate aminotransferase (U/L)	23.43±30.58	18.39±7.38	0.5141
Alanine aminotransferase (U/L)	32.3±60.94	15.84±7.06	0.2766
Albumin (g/L)*	32.14±5.06	41.46±4.78	0.0000
Urea nitrogen (mmol/L)	8.75±6.71	5.36±2.4	0.0808
Serum creatinine (umol/L)	108.53±72.32	71.12±13.4	0.0673
Serum uric acid (umol/L)	374.67±151.8	317.18±108.01	0.2225
Low density lipoprotein (mmol/L)	2.56±1.11	2.33±0.72	0.5010
Total cholesterol (mmol/L)	4.18±1.26	4.2±0.83	0.9540
Triglyceride (mmol/L)	1.73±0.56	1.47±0.95	0.3821
High density lipoprotein (mmol/L)*	0.89±0.38	1.32±0.32	0.0022

Note: *p < 0.05

## Abbreviations

ALT: alanine aminotransferase
AST: aspartate aminotransferase
BCA: bicinchoninic acid
CCN1: communication network factor 1
CYP61: cytochrome P450 61
Db-HFD: Db mice-high fat diet
DEGs: differentially-expressed genes
DFUs: diabetic foot ulcers
DM: diabetes mellitus
ECL: enhanced chemiluminescence
ECM: extracellular matrix
HDL-C: high-density lipoprotein cholesterol
H&E: hematoxylin and eosin
IL1β: interleukin 1β
IP: intraperitoneal injection
LDL-C: low-density lipoprotein cholesterol
nDFU: non-diabetic foot ulcer
LEA: lower-extremity amputation
PMSF: phenylmethanesulfonyl fluoride
PVDF: polyvinylidene difluoride
RBC: red blood cells
RT-qPCR: reverse transcription polymerase chain reaction
SA-β-gal: senescence-associated beta-galactosidase
SASP: senescence-associated secretory phenotype
SDS-PAGE: sodium dodecyl sulfate-polyacrylamide gel electrophoresis
SnCs: senescent cells
TC: total cholesterol
TNFα: tumor necrosis factor alpha
VEH: vehicle
WBC: white blood cells
WT-HFD: wild-type high-fat diet
WT-ND: wild-type normal diet
WT: wild-type
