# Supplementary material for: Clearance of senescent cells enhances skin wound healing in type 2 diabetic mice
Source: Theranostics. 2024 Aug 26;14(14):5429–42. doi: 10.7150/thno.100991 (PMC11413796; doi:10.7150/thno.100991)
Supplement: Supplementary file 1 — Supplementary figures. [file thnov14p5429s1.pdf]

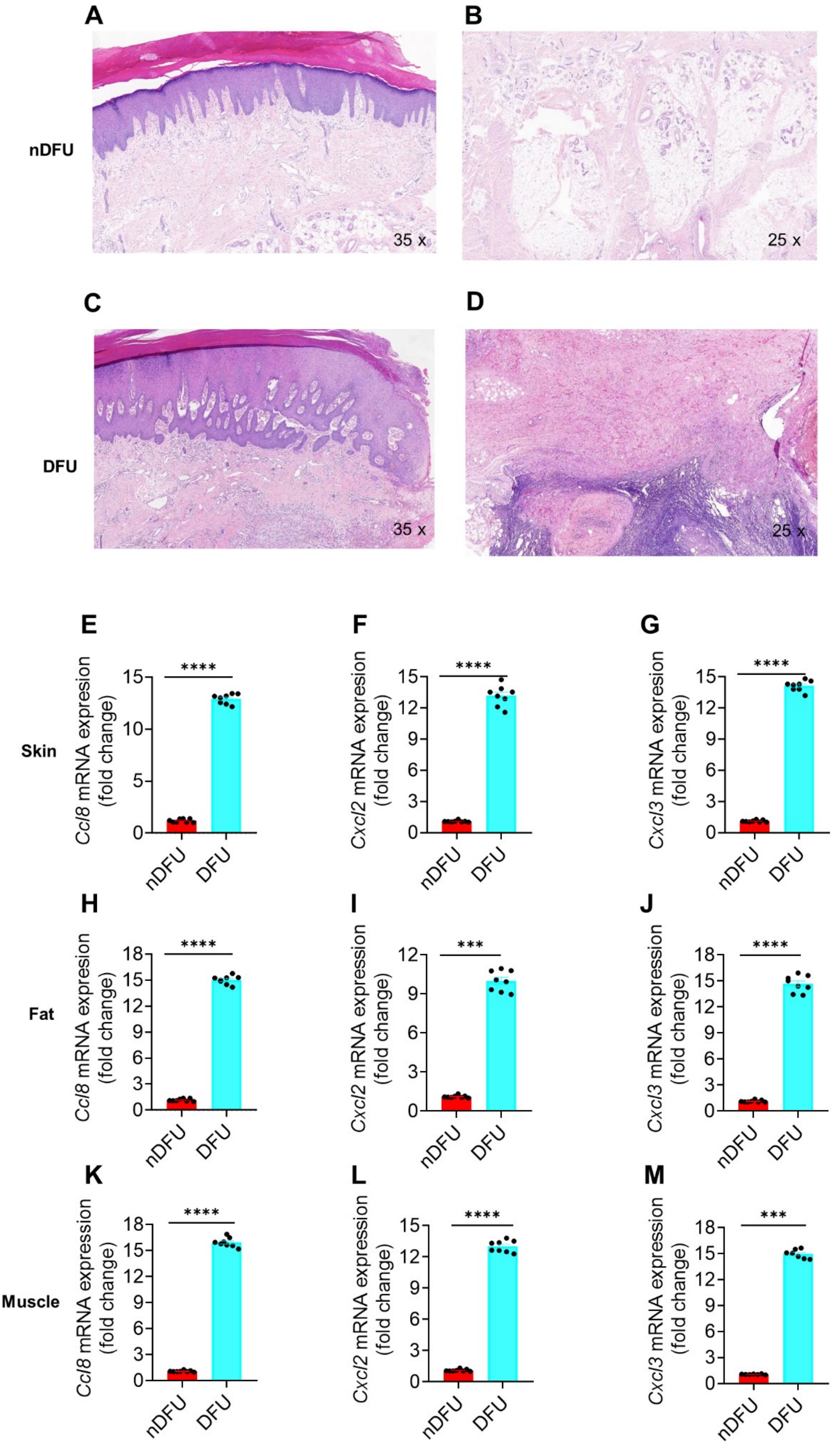
A
B
nDFU
35 x
25 x
C
D
DFU
35 x
25 x
E
F
G
Skin
Ccl8 mRNA expresion (fold change)
Cxcl2 mRNA expresion (fold change)
Cxcl3 mRNA expresion (fold change)
****
****
****
nDFU
DFU
H
I
J
Fat
Ccl8 mRNA expresion (fold change)
Cxcl2 mRNA expresion (fold change)
Cxcl3 mRNA expresion (fold change)
****
***
****
nDFU
DFU
K
L
M
Muscle
Ccl8 mRNA expresion (fold change)
Cxcl2 mRNA expresion (fold change)
Cxcl3 mRNA expresion (fold change)
****
****
***
nDFU
DFU

Fig. S1. Representative pathological staining and senescence markers in the wounds of patients with non-diabetic foot ulcers (nDFU) and diabetic foot ulcers (DFU). (A, B) HE staining of nDFU skin. (C, D) HE staining of DFU patient skin. (E-G) SASP factors (*Ccl8, Cxcl2, Cxcl3*) in the wound skin were detected by qPCR (n=6 for each group). (H-J) SASP factors in the wound fat were detected by qPCR (n=6 for each group). (K-M) SASP factors in the wound muscle were detected by qPCR (n=6 for each group). Data were analyzed by two-sided Student's t-test and presented as Mean ± SEM. ***$p< 0.001$, and ****$p< 0.0001$ compared to nDFU group.

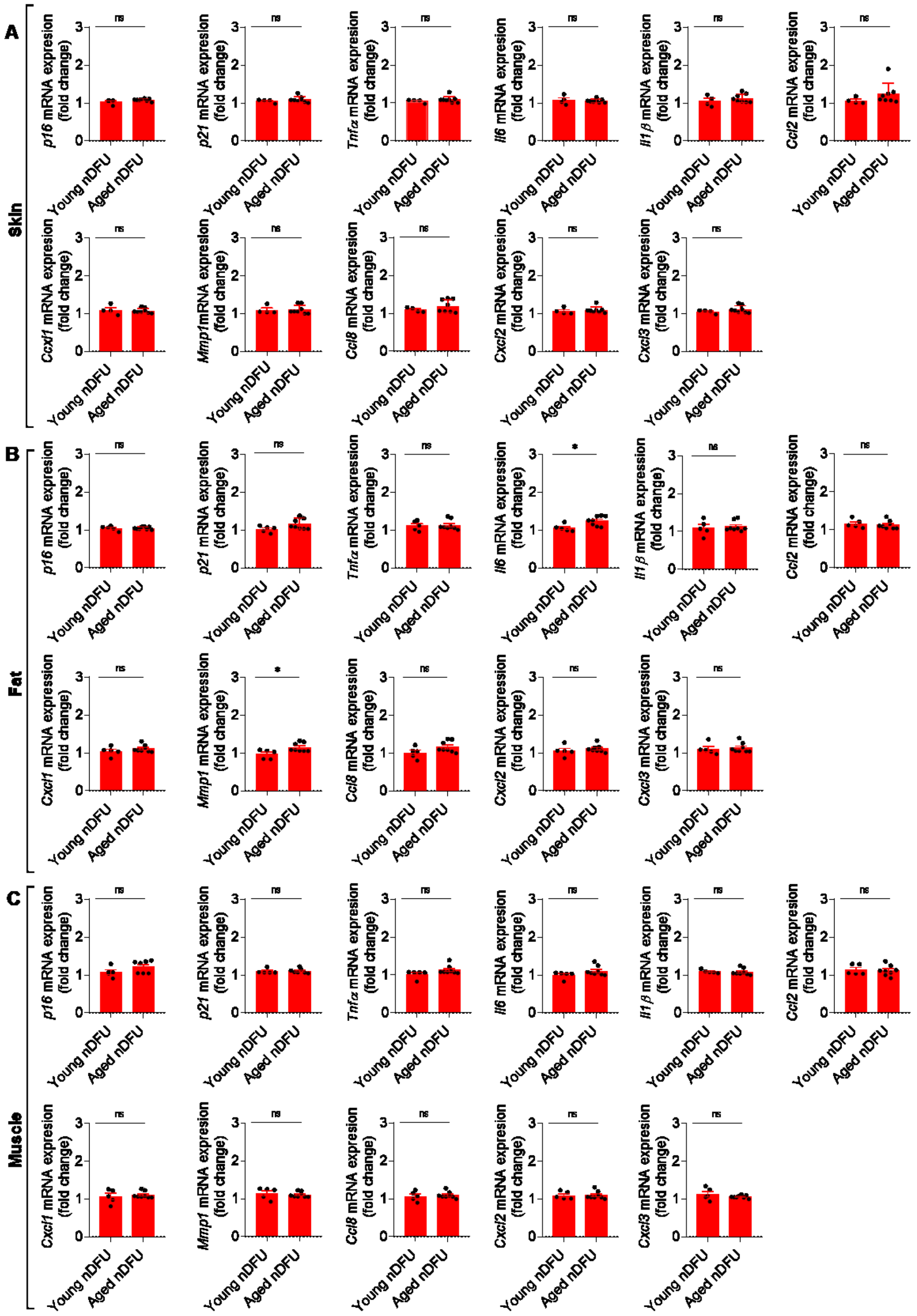

Fig. S2. Gene expression of p16, p21, and SASP factors in the skin, fat, and muscle tissues of younger (35-50 years, n=5) and older non-diabetic foot ulcer (nDFU) patients (>55 years, n=8). Data are presented as Mean ± SEM and were analyzed using a two-tailed Student's t-test. *p<0.05 compared to the younger nDFU group; ns, not significant.

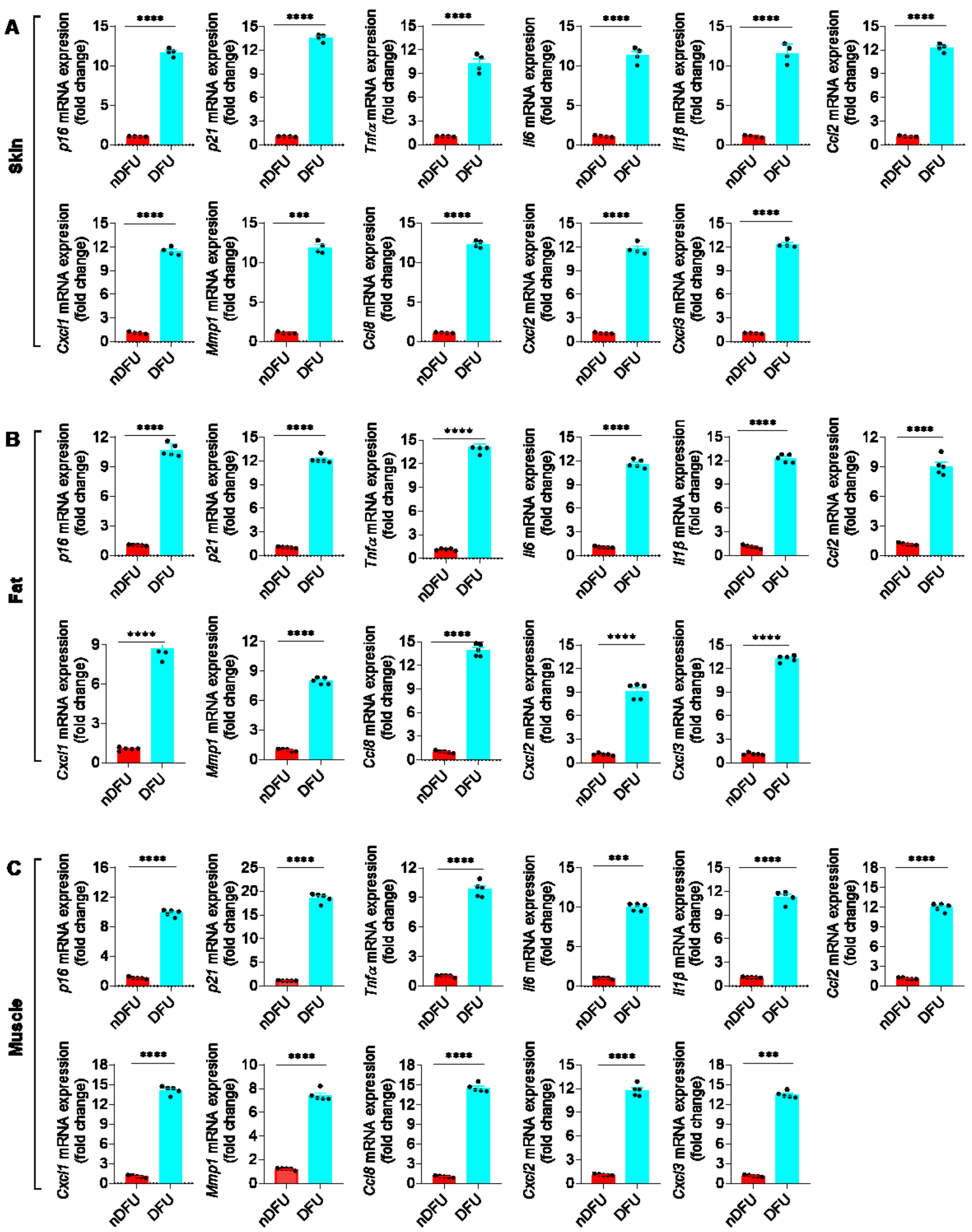

Fig. S3. Gene expression of p16, p21, and SASP factors in the skin, fat, and muscle tissues of young non-diabetic foot ulcer (nDFU) and diabetic foot ulcer (DFU) patients (ages 35-50, n=4 for each skin tissue group, and n=5 for each fat and muscle tissue group). Data are presented as Mean ± SEM and were analyzed using a two-tailed Student's t-test. ***$p< 0.001$, ****$p< 0.0001$ compared to the nDFU group.

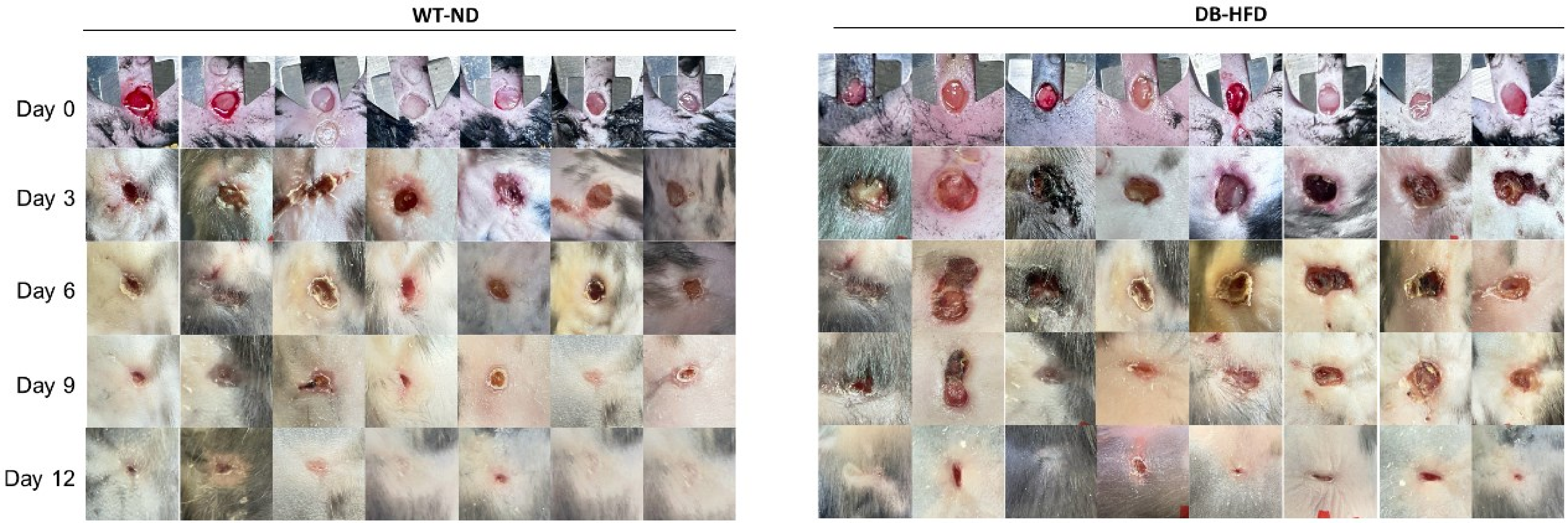

Fig. S4. Wound healing process in mice. The dorsal skin of WT-ND and Db-HFD mice was wounded using 6-mm punches, followed by a scale of the wound area at the indicated time points (n=7 for each group). WT-ND, wild-type mice fed a normal diet; Db-HFD, db mice fed a high-fat diet.

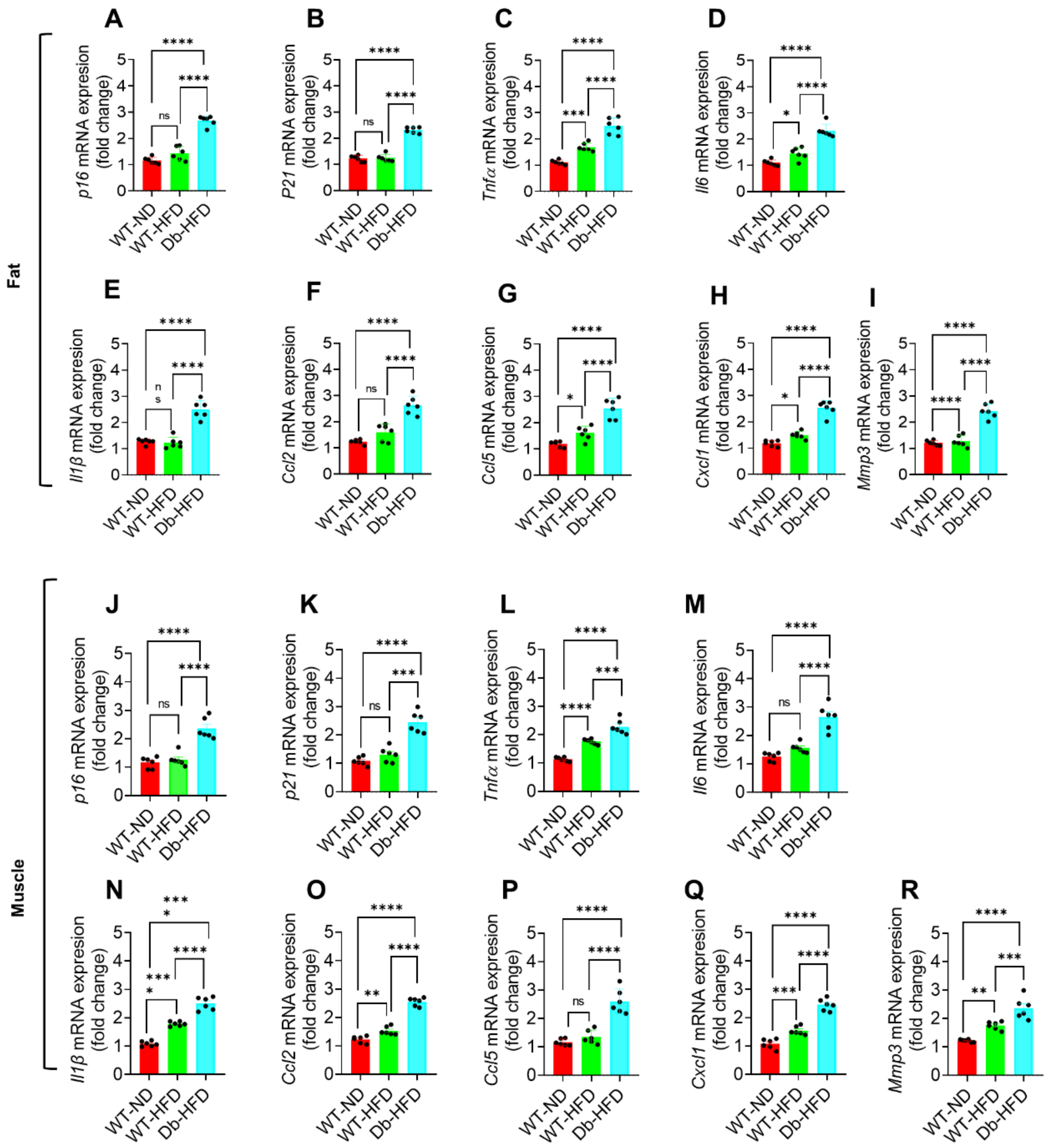

Fig. S5. Accumulation of senescent cells (SnCs) at the wound sites of mice. The dorsal skin of mice was wounded using 6-mm punches, followed by a scale of the wound area at the indicated time points (n=7 for each group). (A-I) mRNA levels of *p16*, *p21*, and SASP factors (*Tnfα, Il6, Il1b, Ccl2, Ccl5, Cxcl1, and Mmp1*) in wound fat. (J-R) mRNA levels of *p16*, *p21*, and SASP factors in wound muscle. 6 mice from each group were randomly selected for senescence marker detection. Data were analyzed by two-sided Student's t-test and presented as Mean ± SEM. *p<0.05, **p<0.01, ***p<0.001, and ****p<0.0001 in the indicated comparisons. WT-ND, wild-type mice fed a normal diet; WT-ND, wild-type mice fed a high-fat diet; Db-HFD, db mice fed a high-fat diet.

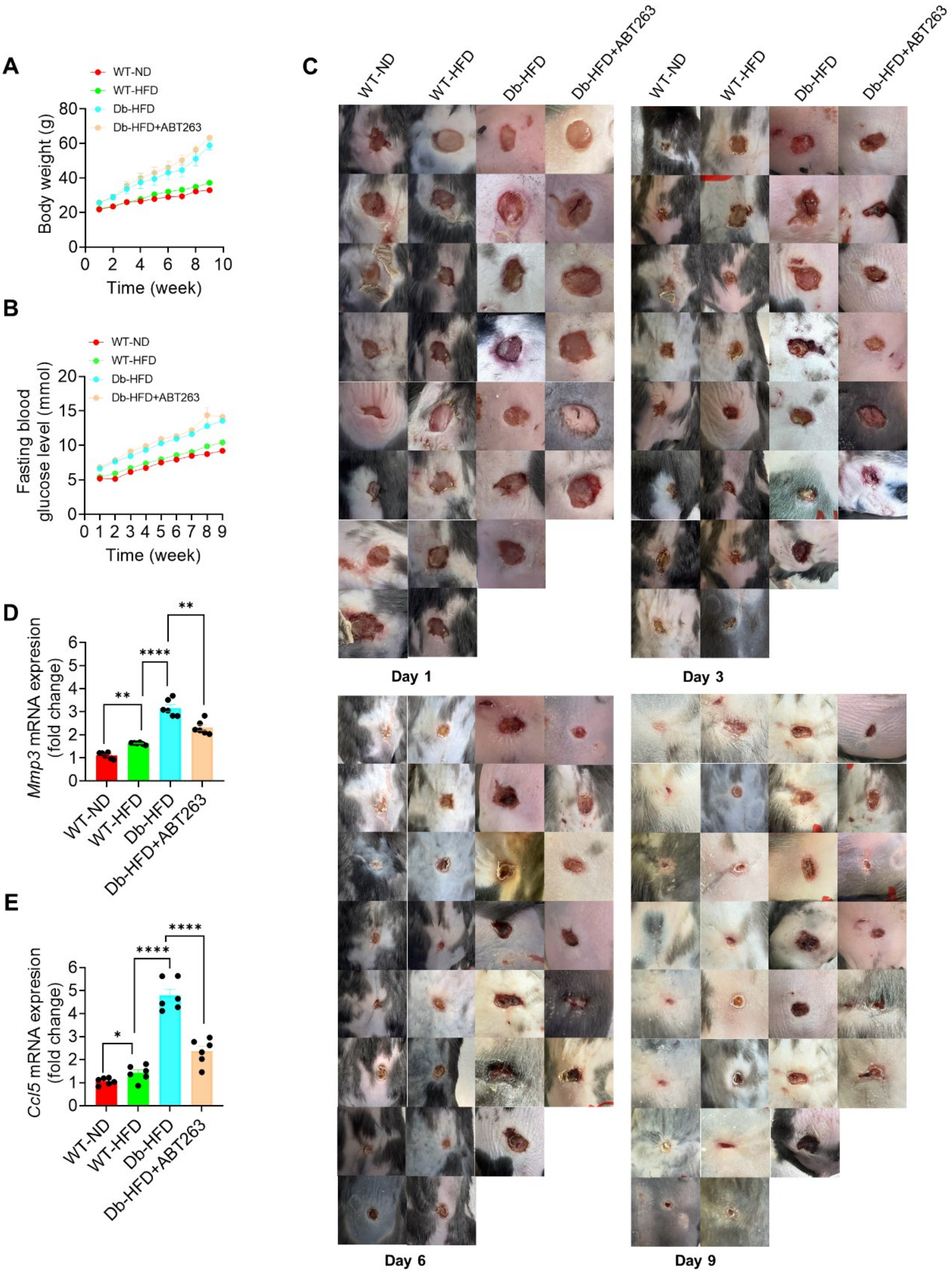

Fig. S6. Clearance of senescent cells (SnCs) with ABT263 accelerated wound healing in diabetic mice. (A) Body weight and (B) fasting blood glucose levels in WT-ND and Db-HFD mice. (C) Images for wound area in the mice of each group at the indicated time points (n=8, 8, 7, 6 for WT-ND, WT-HFD, Db-HFD and Db-HFD+ABT263 groups, respectively. (D, E) SASP factors (*Mmp3, Ccl5*) in wound skin (n=6 for each group). The data are analyzed by one-way ANOVA and presented as Mean ± SEM. *p<0.05, **p<0.01, ***p<0.001 and

****$p<0.0001$ in the indicated comparisons. WT-ND, wild-type mice fed a normal diet; WT-ND, wild-type mice fed a high-fat diet; Db-HFD, db mice fed a high-fat diet.
